# Spectrum feature extraction method combining Allan variance, VMD, and PSD

**DOI:** 10.1038/s41598-024-61176-2

**Published:** 2024-05-14

**Authors:** Xu Liu, Jian Wang, Fei Liu, Craig Hancock

**Affiliations:** 1 School of Geomatics and Urban Spatial Informatics, Beijing University of Civil Engineering and Architecture, Beijing, 102616 China; 2 School of Architecture, Building and Civil Engineering, Loughborough University, Loughborough, Leicestershire LE11 3TU UK

**Keywords:** Allan variance, Variational modal decomposition (VMD), Power spectral density (PSD), Spectrum feature extraction, Micro-seismic event, Natural hazards, Civil engineering

## Abstract

Spectrum feature extraction plays a crucial role in identifying seismic events and calculating structural response parameters. However, the criteria for identifying effective modal components in Variational Mode Decomposition (VMD) are not well-defined, resulting in inaccurate spectrum feature extraction. To address this issue, we propose a novel spectrum feature extraction method that combines Allan variance, VMD, and power spectral density (PSD). Firstly, VMD is applied to filter noise components from triaxial accelerometer observations and add effective signals. Secondly, PSD is utilized to extract three groups of seismic frequencies (tri-axial accelerometers). Finally, the Allan method is introduced to identify the group of accelerometer observations with the highest reliability as the vibration frequency caused by the seismic excitation. We validate the effectiveness of our method by analyzing a Mw 2.6 micro-seismic event that occurred in Huairou, Beijing in 2022. The result shows that our proposed method accurately extracts spectrum features of the Great Wall. Specifically, the seismic excitation vibration frequencies at four monitoring stations were found to be 26.95 Hz, 12.89 Hz, 12.89 Hz, and 12.5 Hz. These findings underscore our method's utility in evaluating the Great Wall's structural response to seismic loading, which has significant implications for the conservation and protection of heritage structures.

## Introduction

China experiences a high frequency, intensity, and distribution of earthquakes, which accounts for one-third of the world’s terrestrial earthquakes and over half of the global earthquake related death toll^1^. Long-term monitoring of seismic activity is crucial for earthquake early warning, prediction, and disaster prevention^2,3^.

The proper extraction of spectral features is crucial in earthquake event recognition as the core component of seismic monitoring^4^. Power Spectral Density (PSD) is commonly employed to extract earthquake signal frequencies^5^. However, the presence of disturbances such as high-frequency noise can result in PSD containing multiple peaks, thus making it challenging to identify the vibration signal frequency generated by earthquake excitation^6^. In recent years, Variational Mode Decomposition (VMD) has gained popularity as a signal denoising method^7^. VMD decomposes a signal layer by layer from high to low frequency, removes noise mode components based on noise component discrimination criteria, and adds up the remaining effective mode components to obtain the denoised reconstruction signal^8^. The key to effectively removing noise using VMD lies in the noise component discrimination criterion^9^. Flandrin proposed using the numerical mutation point of the product of the energy density and average period of modal components to judge noise components^10^. When the numerical mutation point occurs, the first n − 1 modal components are considered noise components, and the following n − k modal components contain useful signal as effective mode components^11^. However, practical applications may present multiple numerical mutation points or unclear mutation points, leading to incomplete noise filtering or even erroneous removal of useful signals. Even after incomplete noise filtering, spectrum analysis results may exhibit multiple peaks. A potential solution is to use three-axis accelerometers, which can monitor earthquake signals, and utilize the same characteristic frequency extracted from all three axes for identifying the frequency generated by earthquake events.

Allan variance is a method of analyzing time-domain signals originally developed by David Allan in the 1960s to assess the frequency and phase stability of oscillators^12,13^. Given the similarities between accelerometers and oscillators, this methodology has become a commonly employed tool recognized by the IEEE for accelerometer parameter analysis^14^. While Allan variance is most effectively used for examining static data, it should be noted that its utility may be limited for high-dynamic data, such as seismic events. Nevertheless, the technique can serve as a supplementary approach for assessing data reliability.

To address the problem of PSD spectra containing multiple peaks, making it difficult to determine the true vibration frequency of the signal, we utilized the VMD denoising method. However, this method only removes a portion of the high/low-frequency noise, while the noise at intermediate frequencies remains mixed with the valid signal. As a consequence, the PSD spectrum still contains multiple peaks. When performing spectral analysis using triaxial acceleration data, the observed shared peak can be regarded as the frequency peak of the effective signal. However, it is worth noting that the accuracy and reliability of three-axis accelerometers can vary. To assess their effectiveness, the Allan variance method can be employed to assess their effectiveness.

In this paper, we present a novel spectral feature extraction method that integrates Allan variance, VMD, and PSD. Firstly, VMD is applied to eliminate the noise component of the three sets of acceleration signals collected by the triaxial accelerometers. Then, PSD is performed to extract vibration frequencies of each set of acceleration signal. If the three power spectrums have the common peak frequency, which can be as the seismic vibration frequency. If there are no common peak frequencies among the three power spectrums, Allan variance analysis is employed on each set of signals to find the highest reliability signal and its vibration frequency can be as the final seismic vibration frequency. The proposed approach is evaluated using observational data obtained from three-axis accelerometers installed at four monitoring stations along the Great Wall in Huairou, Beijing. Our findings indicate that the proposed methodology is robust and dependable in terms of accurately identifying characteristic frequencies associated with seismic events. Overall, our results suggest that this method is highly effective and has strong potential for practical applications in the field of conservation and protection of heritage structures.

## A spectrum feature extraction method for earthquakes combining Allan variance, VMD and PSD

### Allan variance

The Allan variance time-domain analysis method was created by David Allan in the 1960s to assess oscillators’ frequency and phase stability^15^. This approach has gained widespread recognition from IEEE for its ability to analyze gyroscope and accelerometer parameters due to similarities between these devices and oscillators. By effectively isolating various noise sources, it can accurately identify each one's characteristic parameters. The Allan variance has two primary applications: the first involves evaluating gyroscope and accelerometer performance, providing valuable insights for hardware grouping, parallel schemes, and user selection of inertial devices. Secondly, it determines noise parameters in random error modeling.

Assuming that a set of data is continuously collected at a time interval $\tau_{0}$, the data is grouped in sequential $y_{i} ,i = 1,2,...N$ order according to the time series with each group consisting of m sampling points($m < N/2$, the value of *m* is usually $2^{0} ,2^{1} ,2^{2} ,...2^{i} ,\;\;i = (\lg N/\lg 2) - 1$). The average duration (time cluster) of each group is $m\tau_{0}$, which is divided into *K* groups. The steps for calculating Allan variance are as follows^16^:

(1) Calculate the mean value of each group of data.

1 $$Y_{k} = \frac{1}{m}\sum\limits_{n = k}^{k + m - 1} {y_{i} } ,\quad k = 1,2,...N - m + 1$$

(2) Calculate the difference between adjacent groups of data.

2 $$D_{k} = Y_{k + 1} - Y_{k} ,\quad k = 1,2,...N - 2m + 1$$

(3) Standard Allan variance formula:

3 $$\sigma^{2} (\tau ) = \frac{1}{2}\langle (D_{(k - 1)m + 1} + 1)^{2} \rangle ,\quad k = 1,2,...Round((N/m - 1))$$

Overlapping Allan variance formula:

4 $$\sigma^{2} (\tau ) = \frac{1}{2}\langle (D_{k} )^{2} \rangle ,\quad k = 1,2,...N - 2m + 1$$

(4) Plot a double-logarithmic curve, with the horizontal axis representing changes in time clusters and the vertical axis representing the corresponding Allan variance values for each time cluster.

**Figure 1:**
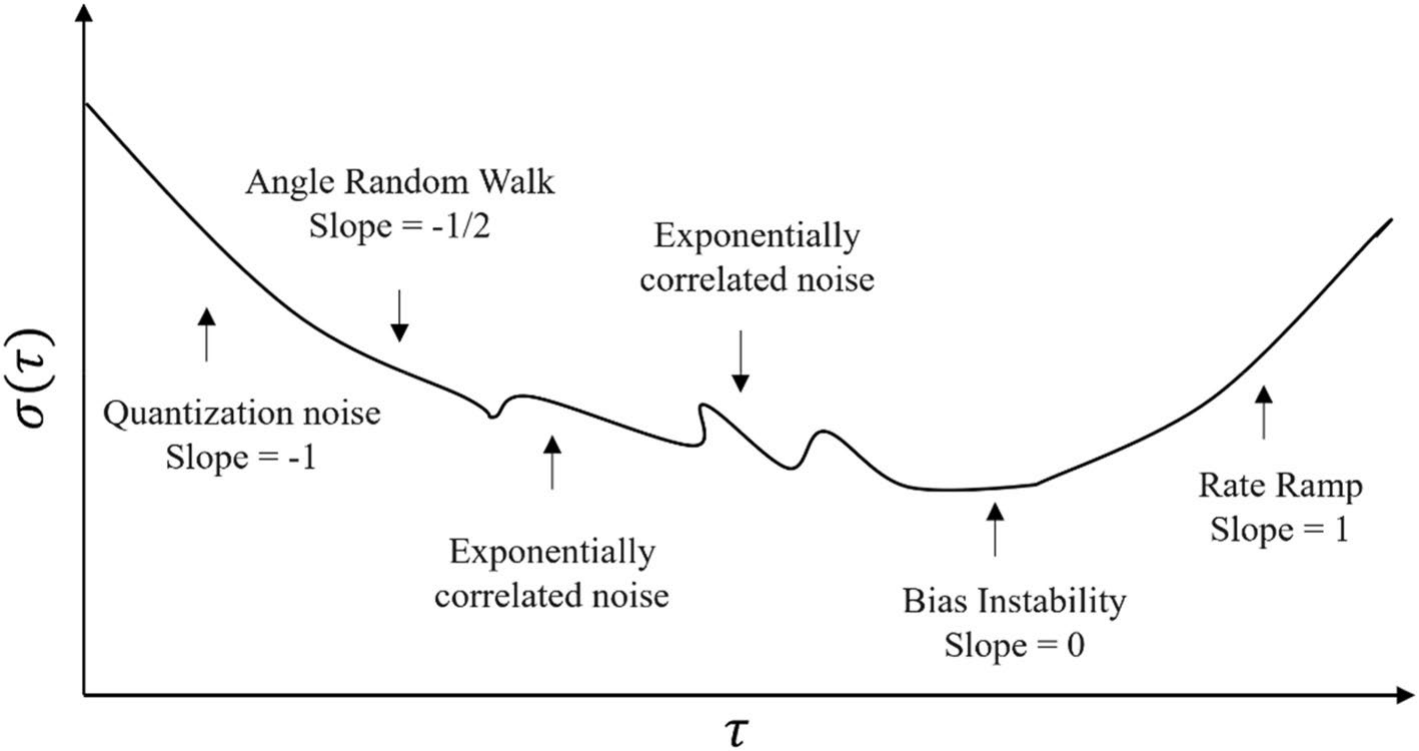
Allan Variance double logarithmic curve.

In Allan variance analysis, we first calculate the noise sequence's Allan variance using Eq. (3). Then, we apply a logarithmic transformation to determine the slope for a specific time $\tau$, creating a double logarithmic curve as seen in Fig. 1. This curve helps identify five noise types: quantization noise, angle random walk, bias stability, rate random walk, and rate ramp. By examining the different slopes for each noise type, we can estimate when the errors might occur. Next, we read the intersection points of the tangents for each noise type with the y-axis. Finally, we precisely calculate the error coefficients by relating these intersection readings to the noise coefficients.

### Power spectral density (PSD)

Power spectral density (PSD) is commonly utilised to extract the dominant frequency of random signals through the transformation of a random signal in either the time or space domain to the temporal or frequency domain through the Fourier transform. Power over frequency is shown by the PSD curve, with the horizontal axis representing frequency (Hz) and the vertical axis representing power spectral density (dB/HZ). The calculation process is as follows.

Calculate the Fast Fourier transform (FFT) for the random signal x(n) as a series of finite energies. X(k) is the denominator of the FFT function and is given by

5 $$X\left(k\right)=\sum_{n=0}^{N-1}x(n){e}^{-j2\pi kn/N}$$

N represents the sampling points for $x(n)$, j represents the imaginary number, $k=\mathrm{0,1},\dots ,N-1$

While FFT reveals the frequency composition of a signal, PSD further provides insights into the power present in each frequency component of the signal. This proves especially useful in contexts where it's crucial to comprehend how energy or power is distributed across frequencies, such as in vibration analysis^21^.

Estimate the PSD by dividing the modular square of X(k) by “N”. The PSD formula can be expressed as follows.

6 $$S\left(k\right)=\frac{1}{N}{\left|X\left(k\right)\right|}^{2}$$

Due to the large variance of the estimates, the traditional PSD mentioned above is not effective. In addition, the variance does not decrease as the x(n) length increases (Wang 2006). To overcome these limitations, Welch’s Method proposed the Averaged Periodogram (Welch’s Method) by splitting the random signal into segments with partial overlap between each segment. After that, the PSD for each segment is calculated. Finally, divide it by the average.

The function of Averaged Periodogram is defined as:

7 $${S}_{xx}\left(k\right)=\frac{1}{{MN}_{FFT}}\sum_{i=1}^{M}{X}_{i}(k){X}_{i}^{*}(k)$$

where ${X}_{i}(k)$ is the Fourier transform of the i-th segment of a random signal, ${X}_{i}^{*}(k)$ is the Conjugate plural of ${X}_{i}(k)$, M is the average number of times^21^.

### A denoising method combining VMD with white noise characteristics

Variational Mode Decomposition (VMD) is a method for decomposing signals layer by layer from high to low frequency. It is essentially an enhanced version of the Empirical Mode Decomposition (EMD) algorithm but resolves the mode mixing issue prevalent in EMD. Consequently, VMD has increasingly replaced the EMD algorithm in recent years^9^.

**Figure 2:**
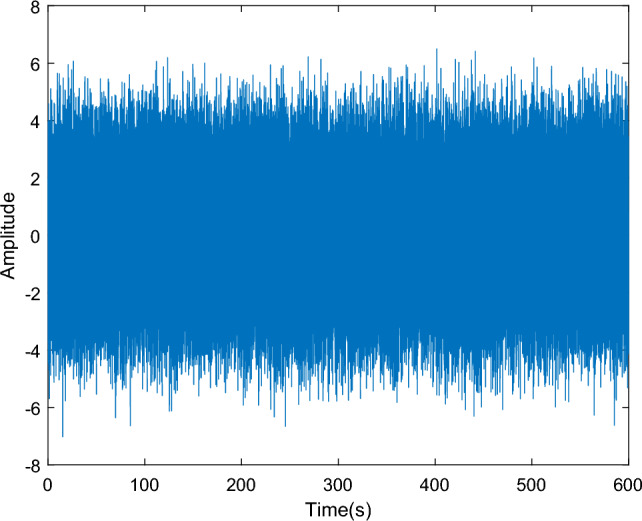
Analog signals.

**Figure 3:**
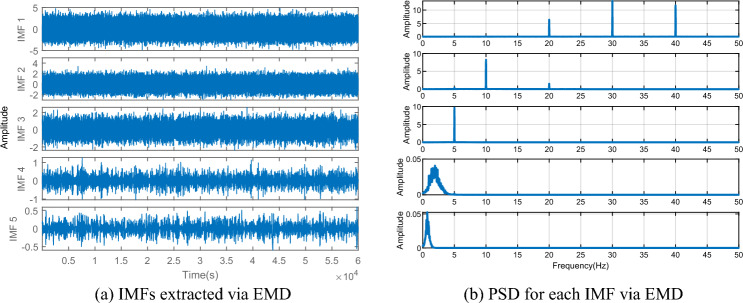
IMFs extracted via EMD and PSD for each IMF.

**Figure 4:**
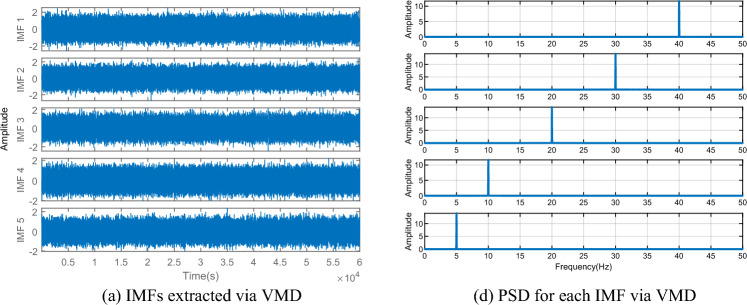
IMFs extracted via VMD and PSD for each IMF.

To verify the superiority of the VMD algorithm over EMD, a dataset is simulated to compare the performance between traditional EMD and VMD. The simulated data comprises five sinusoidal signals with varying amplitudes and frequencies (5 Hz, 10 Hz, 20 Hz, 30 Hz, and 40 Hz, respectively) sampled at 100 Hz. Random noise is also introduced to the data, as depicted in Fig. 2. After EMD decomposition (see in Fig. 3a), the signals experience frequency aliasing. In Fig. 3b, it can be clearly seen that the PSD curve of IMF1 exhibits three peaks, at 20 Hz, 30 Hz, and 40 Hz, respectively, while IMF2 also displays two peaks, at 10 Hz and 20 Hz. In contrast, VMD effectively separates signals of different frequencies (see in Fig. 4a), and the frequencies of the peaks in the PSD curve correspond to the set frequencies (see in Fig. 4b), indicating that VMD resolves the frequency aliasing issue associated with EMD, thus demonstrating superior performance compared to EMD.

In general, noise has high-frequency properties, and the highest frequency mode after decomposition can be thought of as the signal's high-frequency noise, while the lowest frequency reflects the signal's trend item. Therefore, the conventional method of extracting the effective signal is to remove the high-frequency and low-frequency modes and then reconstruct the signal by summing the remaining models. The expression is as follows:

8 $$\mathrm{X }= \sum_{\mathrm{i }= 1}^{{\text{n}}}{{\text{IMF}}}_{{\text{i}}}$$

9 $$X^{\prime } = X - IMF_{1} - IMF_{n}$$

In the equation, X represents the original signal, IMF represents the decomposed modes, n indicates the maximum level of decomposition, and X′ represents the useable signal. This method, however, cannot accurately extract the usable information of the signal since there are residual components of helpful signals in the highest and lowest frequency signals.

The research findings of Flandrin et al.^10^ indicate that for a Gaussian white noise signal, the product of the energy density of its IMF components and their average period is a constant, which as follows:

10 $${E}_{n}\cdot {\overline{T} }_{n }= const$$

The expression for energy density is:

11 $${E}_{n} = \frac{1}{N}\sum_{j = 1}^{N}{\left[{IMF}_{n}\left(j\right)\right]}^{2}$$

The expression for average period is:

12 $${\overline{T} }_{n} = \frac{N*2}{Count(Optim{a}_{n})}$$

In the equation, $Count(Optim{a}_{n})$ represents the total number of extreme points of the nth mode component ${IMF}_{n}$, and $N$ is the total length of the component's data. Therefore, based on this characteristic of the noise components, The shift points of the product of the IMFs’ modal component’s energy density and average period are identified as useful signal components. The method is subsequently referred to as the Flandrin denoise criterion.

Determining the optimal number of VMD layers is essential. Increasing the number of VMD decomposition layers improves noise isolation but may reduce operational efficiency. This paper proposes a direct and efficient method, initiating with a power spectral density analysis before signal decomposition. This analysis assesses the signal's noise level, allowing for a reduction in decomposition layers to enhance computational efficiency when noise is minimal. Nonetheless, the precise number of layers should be tailored to the engineering efficiency needs, typically recommended to be between 5 and 15.

The denoising process of VMD is as follows: First, the acceleration signal is analyzed by frequency spectrum analysis. A reasonable decomposition level n is set according to the number of spectral peaks. Then, the signal is decomposed by VMD, and the spectrum of each mode is analyzed. Based on the decomposition signal morphology and spectral analysis results, it is determined whether the signal contains a trend term. If it does, the mode component containing the trend term is removed, and the signal is reconstructed. The product of energy density and average period is calculated using the reconstructed signal, and numerical mutation points are regarded as effective signals^17–19^.

### Seismic simulation shaking table experiment

**Figure 5:**
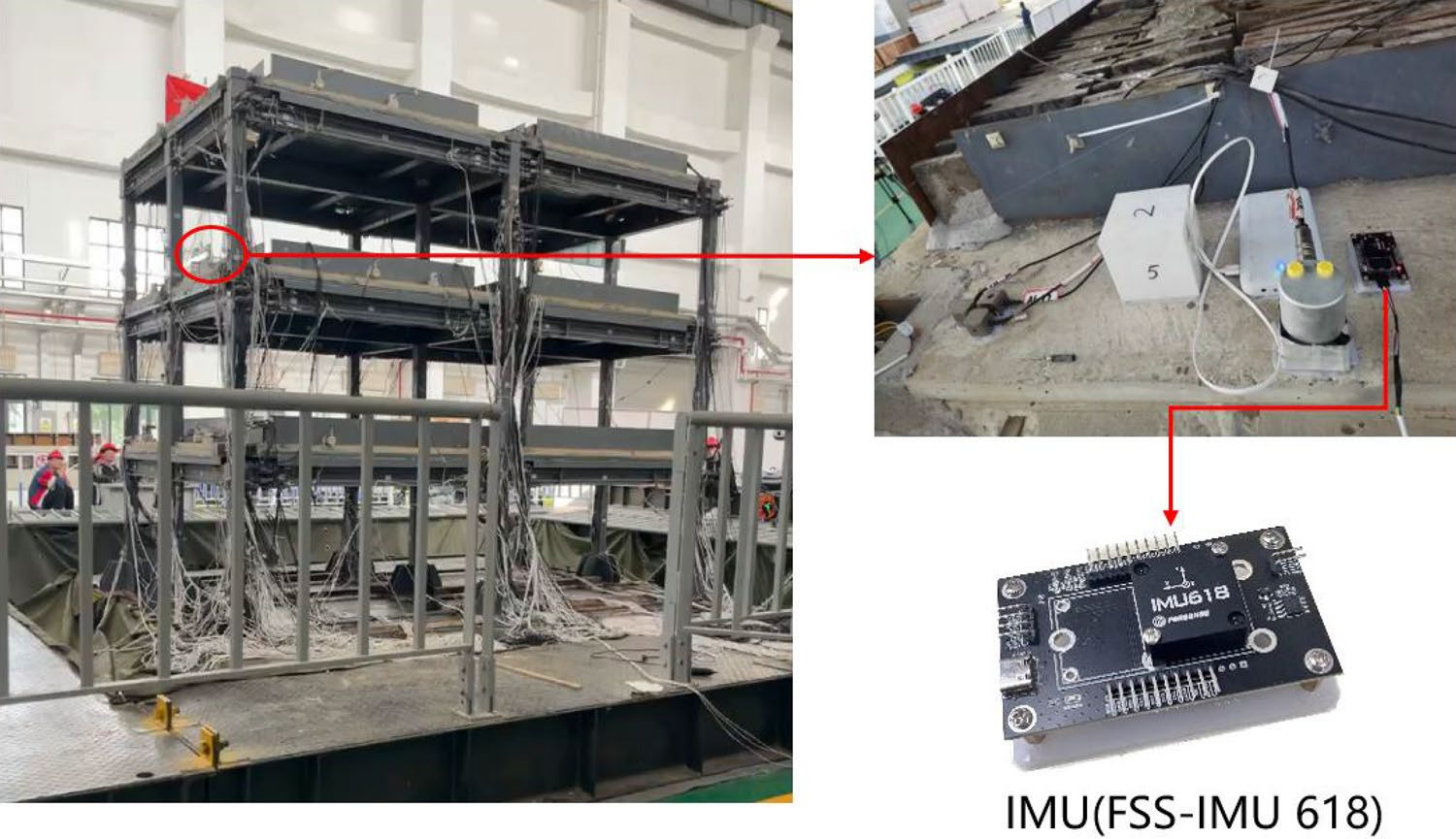
Three-story frame-type steel structure (left) and the layout of IMU which containing a triaxial accelerometer (right).

To validate the proposed denoising method combining VMD with white noise characteristics, a seismic simulation experiment was conducted in Oct 2023 at the multifunctional shaking table array laboratory of Beijing University of Civil Engineering and Architecture. The shaking table is a triaxial six-degree-of-freedom multifunctional seismic simulation platform, capable of carrying loads up to 60 tons, with a table size of 5 × 5 m. It supports a three-story frame-type steel structure (see in Fig. 5left). The triaxial accelerometer used in this experiment is incorporated within the FSS-IMU618 Inertial Measurement Unit (IMU) produced by Forsense, featuring a sampling frequency of 100 Hz, zero-bias instability of 2 μg, and a random walk of 0.03 m/s/√hr. The triaxial accelerometer was securely fixed to the second-floor slab of the frame-type steel structure using solid adhesive (see in Fig. 5right).

The simulated earthquake experiment adopted the EL Centro seismic spectrum, widely used in engineering. The experiment comprised four tests simulating earthquakes with single-axis horizontal seismic peak ground accelerations (PGA) of 0.9 g and 1.1 g. The structure was subjected to horizontal seismic excitation four times, each lasting about 50 s, with details outlined in Table 1.

**Table 1:** Statistics of measurement data of the four tests.

Test	PGA			Duration (s)	Shift Point	Frequencies before denoising	Frequencies after denoising (Hz)
	X	Y	Z				
Test 1	0.9 g	–	–	55	IMF11	2.71 Hz, 7.02 Hz	2.34
Test 2	–	0.9 g	–	51	IMF12	2.34 Hz, 6.26 Hz	2.34
Test 3	1.10 g	–	–	50	IMF11	2.73 Hz, 7.03 Hz	2.34
Test 4	–	1.10 g	–	45	IMF12	2.34 Hz, 6.24 Hz	2.34

**Figure 6:**
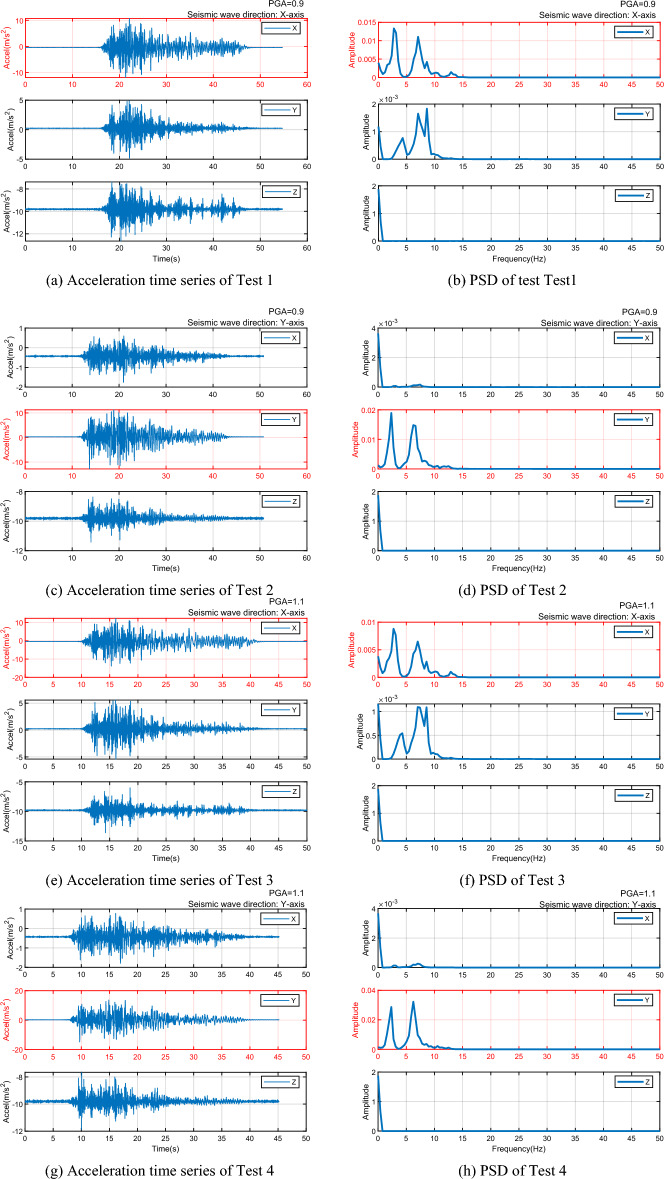
Acceleration time series and PSD of 4 Tests.

The acceleration time series and PSD for the four tests are depicted in Fig. 6. Despite each test being a single-axis vibration, three-axis acceleration time series and corresponding analytical figures were plotted for a comparative analysis. Figure 6a–g shows that in tests with a PGA of 0.9 g, Tests 1 and 2 had maximum acceleration amplitudes of around 11 m/s^2^ (≈1.1 g). In the tests with a PGA of 1.1 g, Test 3’s peak amplitude was about 12 m/s^2^ (≈1.2 g), and Test 4's was 15 m/s^2^ (≈1.5 g), indicating a minor variance between the actual seismic load and the accelerometer's readings, with the most significant discrepancy being 0.3 g (Test 4). Figure 6b–h illustrates that each test had multiple peaks, and even though these peaks were at frequencies close to each other, pinpointing the specific vibration frequency triggered by the earthquake remains difficult.

**Figure 7:**
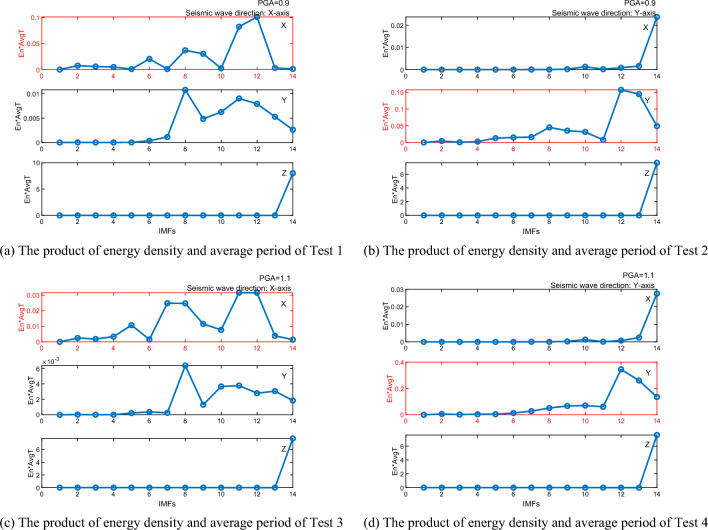
The product of energy density and average period of 4 Tests.

VMD is applied to each signal test, with subsequent calculation of the product of energy density and the average period for each Intrinsic Mode Function (IMF), as shown in Fig. 7. Analysis reveals that for Test 1 (X-axis), the significant change occurs at IMF11 and IMF12, while for Test 2 (Y-axis), it is at IMF12 and IMF13. Test 3 (X-axis) shows a shift at IMF11 and IMF12, and Test 4 (Y-axis) at IMF12. These shift points are pivotal for reconstructing the signal effectively. Notably, the shift points for the non-vibrating axes predominantly occur in IMF14, indicating a low-frequency trend. This suggests that the low-frequency trends significantly affect the non-vibrating axes, potentially overshadowing the actual vibration signals detected.

**Figure 8:**
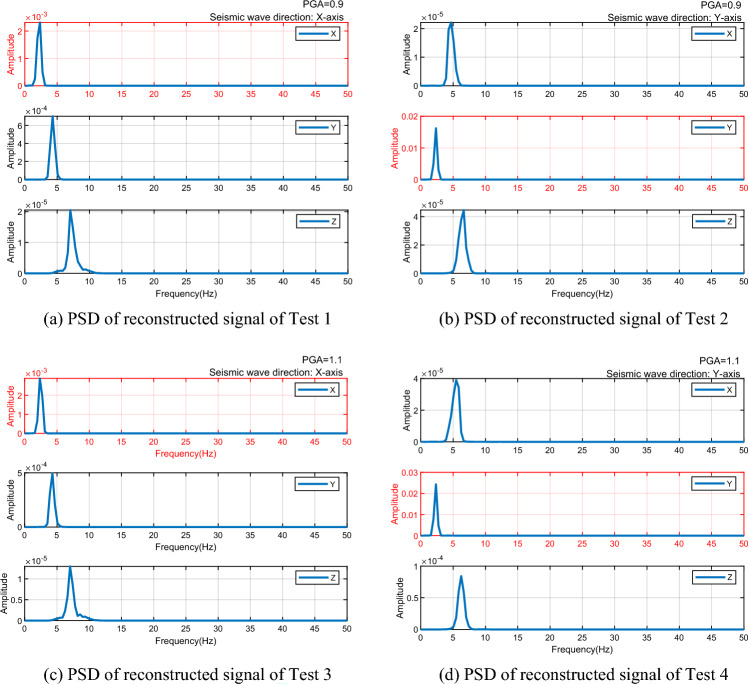
PSD of reconstructed signal of four Tests.

PSD curves of the reconstructed signals are then plotted. The PSD curves for all four tests display a single peak, with the peak frequency consistently at 2.34 Hz, indicating that the vibration frequency of the experimental steel structure is 2.34 Hz (see in Fig. 8). By comparing the frequencies before and after denoising, as presented in Table 1, it becomes evident that the method accurately extracts the vibration frequency of the structure under seismic excitation and effectively eliminates noise from the signal.

### Flowchart for seismic spectral feature extraction method

**Figure 9:**
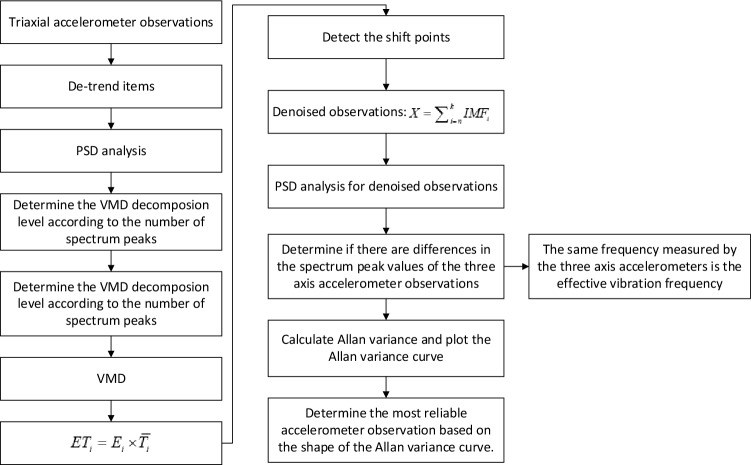
A flowchart for seismic spectrum feature extraction combining Allan variance, VMD, and PSD.

The method for extracting seismic spectrum features, which involves the combination of Allan variance, VMD, and PSD, can be described as follows:

1. Remove the trend items component from the three-axis accelerometer observations and perform frequency analysis to determine the decomposition level n according to the number of spectrum peaks.
2. Employ VMD to decompose the signal into different intrinsic mode functions (IMFs) from the high to low frequency.
3. Calculate the product of energy density (${E}_{i}$) of each IMF with Gaussian white noise and average period (${\overline{T} }_{i}$) of the signal: ${ET}_{i}$=${E}_{i}$×${\overline{T} }_{i}$.
4. Detect the shift points of the product, the shift points and its neighboring modal components are considered as valid signals.
5. Add up the effective modal component signals to obtain the denoised signal.
6. Analyze the frequency spectrum of the signal after denoising to determine if there are differences in the peak values of the three-axis acceleration signals. If there are differences, proceed to step (7); otherwise, the same frequency extracted by the three-axis accelerometer is considered the effective vibration frequency.
7. Calculate the Allan variance and plot the Allan variance curve to determine the most reliable acceleration signal based on the distribution and shape of the Allan variance curve. The frequency measured by this signal is the effective vibration frequency. The flowchart for seismic spectrum feature extraction combining Allan variance, VMD, and PSD is as follows (Fig. 9).

## Trial analysis

### Equipment for monitoring dynamic response under seismic loading

**Figure 10:**
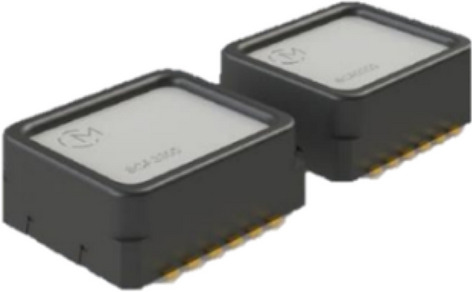
The Great Wall in Huairou district.

**Figure 11:**
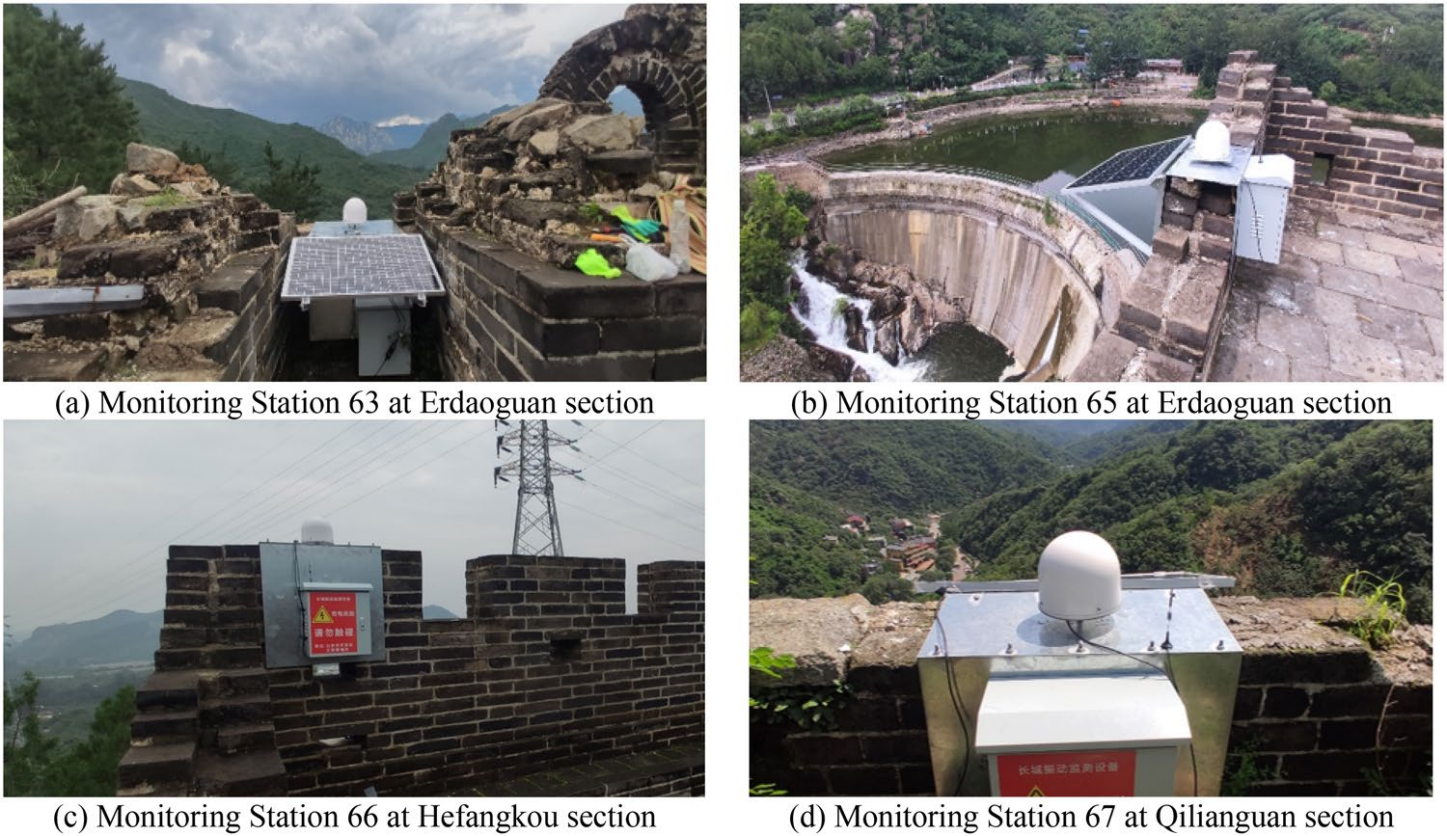
The layout of GNSS/Accelerometer vibration monitoring equipment.

In December 2021, four sets of GNSS/Accelerometer vibration disaster monitoring equipment were installed at the Erdaoguan, Qiliankou, and Hefangkou sections of the Great Wall in Huairou District. This setup includes an SCA3300 model accelerometer from Murata, a UB4B0M GNSS board card by Unicore Communications, GNSS and 4G communication antennas, a solar power supply panel, and a waterproof enclosure. The equipment is operational, and this paper focuses exclusively on the data from the triaxial accelerometers, specifically the SCA3300 model with a sampling frequency of 100 Hz (see in Fig. 10). The specifications of this model are detailed in Table 2. Before installation, the accelerometer had already undergone factory calibration and laboratory calibration. The Monitoring Station 63 and Station 65 accelerometers are located at the Erdaoguan section, the Station 67 accelerometers are at the Qilianguan section, and the Station 66 accelerometers is at the Hefangkou section, with the deployment position of the equipment as shown in Fig. 11.

**Table 2:** Principal specifications of the triaxial accelerometers.

Performance index	Parameter
Measurement range	6 g
Noise density	37 $\mathrm{\mu g}/\sqrt{{\text{Hz}}}$
Offset error	1.15 mg
Linearity error	1 mg
Initial bias error (1 year)	10 mg
Sampling frequency	100 Hz

### Micro-seismic events in Beijng

#### A Mw 2.6 micro-seismic event in Huairou, Beijing in 2022

**Figure 12:**
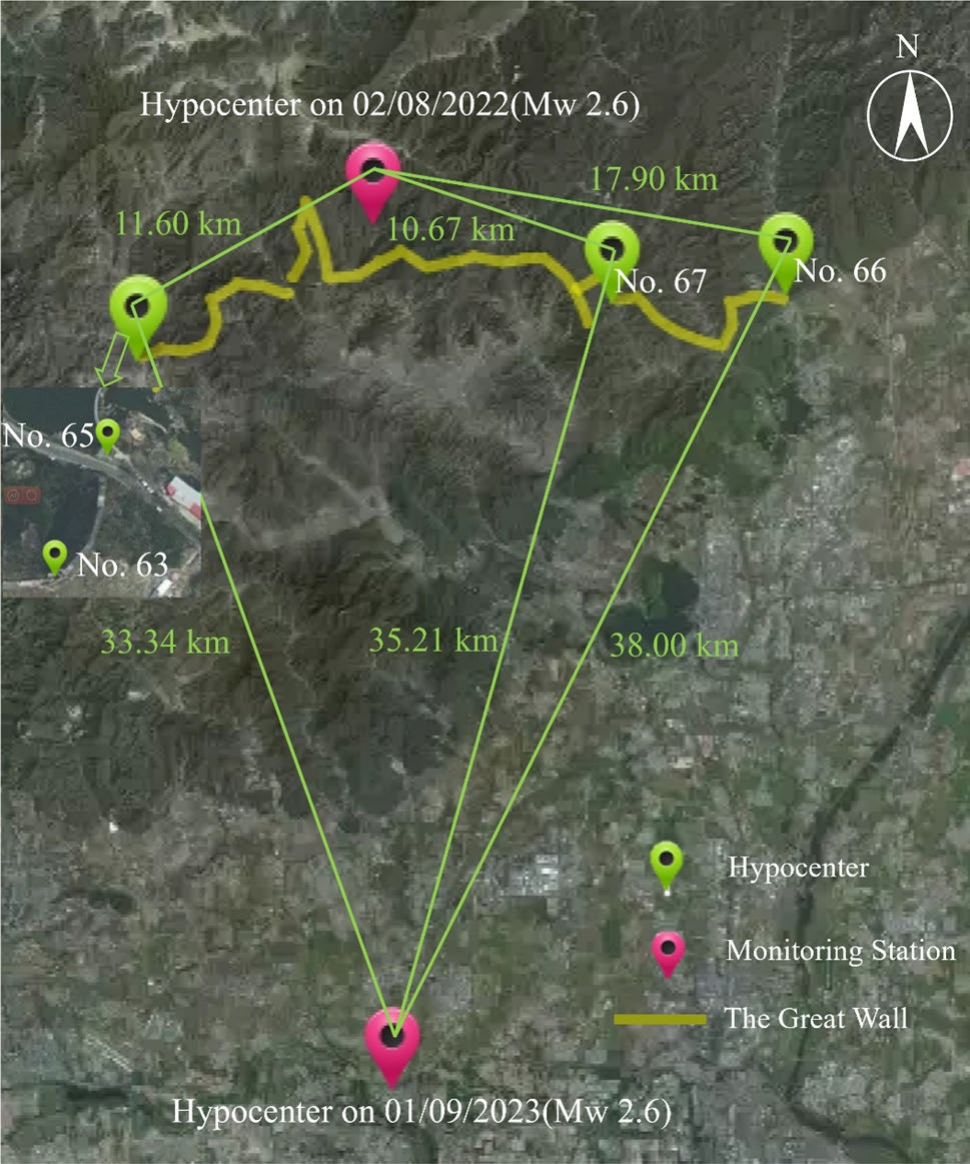
Hypocenters and monitoring stations. (Drawn based on Ovital map: https://www.ovital.com/).

On August 2, 2022 at 06:31, a magnitude 2.6 earthquake occurred in Huairou District, Beijing, at a focal depth of 18 km and an hypocenter located at 40.48 degrees North latitude and 116.47 degrees East longitude, as reported by the China Earthquake Networks Center. The hypocenter of this micro-seismic event was 11.92 km away from monitoring station 63, 11.60 km from station 65, 17.90 km from station 66, and 10.67 km from station 67 (see in Fig. 12).

#### A Mw 2.7 micro-seismic event in Changping, Beijing in 2022

On September 1, 2023, at 15:34, a magnitude 2.7 earthquake struck Shunyi District of Beijing. The earthquake had a focal depth of 17 km and was located at a latitude of 40.14 degrees north and a longitude of 116.48 degrees east. The hypocenter was 32.26 km from station 63, 33.34 km from station 65, 35.21 km from station 66, and 38 km from station 67. The two hypocenters are approximately 36.83 km apart (see in Fig. 12).

The two earthquakes were categorized as micro-seismic events, exhibiting a moment magnitude (Mw) between 1 and 3, consistent with the conventional definition of microseisms. Although such micro-earthquakes are typically too weak to inflict damage on engineering structures or cause casualties or property loss, they possess the potential to induce resonance or structural loosening owing to their low natural frequencies. Additionally, they may cause some level of impact on subterranean rock and soil structures.

### Acceleration time series analysis

**Figure 13:**
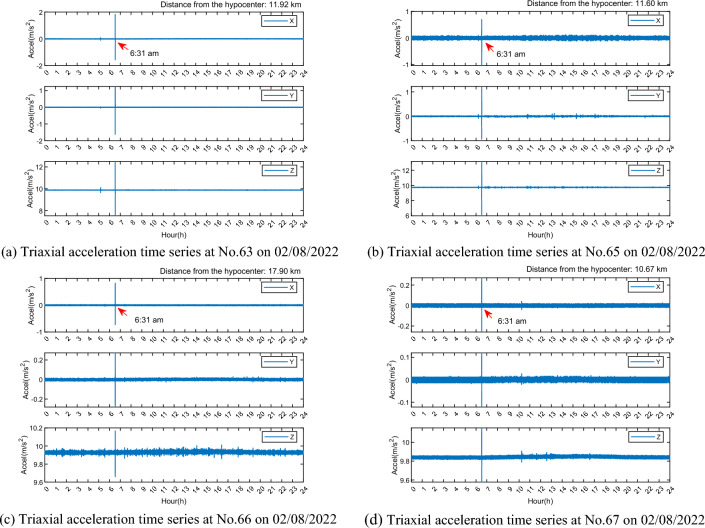
Acceleration time series of four monitoring stations on 02/08/2022.

To identify the earthquake event and evaluate its impact on accelerometer data, the data from four monitoring stations were downloaded and analyzed. For data on August 2, 2022, the acceleration time series data shows that significant vibrations occurred simultaneously at all four stations at 06:31:55 Beijing time (23,515 s), which coincides with the earthquake occurrence time. The amplitude of the acceleration time series of station 63 is the highest, approaching 2 m/s^2^, followed by station 65, 66, and 67 (see in Fig. 13). This indicates that there is no correlation between the amplitude and the distance from the hypocenter. It is speculated that the lack of a clear correlation is due to the relatively minor differences in distances between each monitoring station and the hypocenter. Additionally, various factors such as geological structures and the direction of seismic waves contribute to the absence of a significant correlation between distance and amplitude.

**Figure 14:**
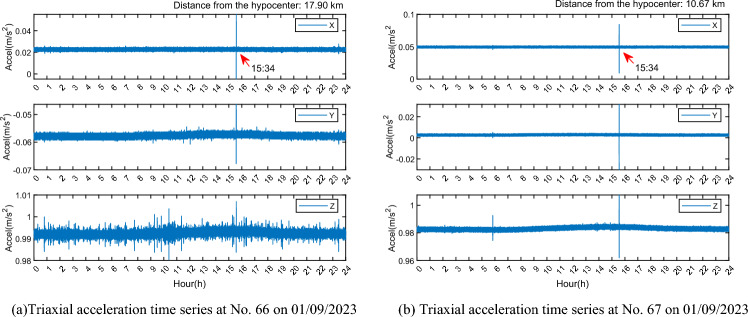
Acceleration time series of two monitoring stations on 01/09/2023.

To explore the impact of the Shunyi, Beijing earthquake on the Great Wall structure, researchers analyze acceleration data from September 1, 2023. As monitoring stations 63 and 65 are offline on that day, they exclusively utilize the acceleration data from stations 66 and 67. The time series data show that the amplitude at station 67 slightly exceeds that at station 66, with the maximum amplitude approximately 0.4 m/s^2^ (see in Fig. 14). This amplitude is an order of magnitude smaller than that observed during the minor tremor in Huairou, Beijing, on August 6, 2022. The discrepancy arises because the monitoring stations are closer to the hypocenter of the 02/08/2022 event and farther from the hypocenter of the 01/09/2023 event.

The acceleration time series from the two micro-seismic events indicate that the triaxial accelerometer can detect micro-seismic activities, which have a discernible impact on the structure of the Great Wall. The degree of impact is correlated with the proximity to the events. Due to space constraints, the following analysis will focus solely on the data from the micro-seismic event closer to the epicenter, which occurred in Huairou, Beijing, on August 2, 2022.

### Spectrum analysis

**Figure 15:**
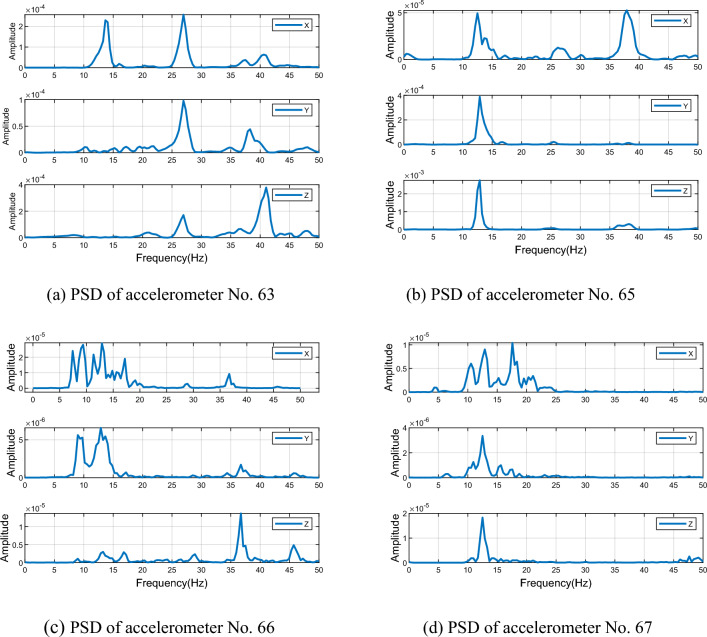
PSD of accelerometer.

To determine the vibration frequency resulting from earthquake excitation, the power spectral density (PSD) of X, Y, and Z directions was analyzed for all four monitoring stations. Figure 15 displays the PSD curves of the three-axis acceleration time series during the earthquake period at these stations, revealing variations in spectral peak values among the X, Y, and Z directions, along with multiple spectral peaks on each axis. These differences stem from gross errors and disturbances in the accelerometer time series, which pose challenges in identifying the vibration frequency caused by the earthquake excitation. Consequently, error separation and noise reduction processing are required.

**Figure 16:**
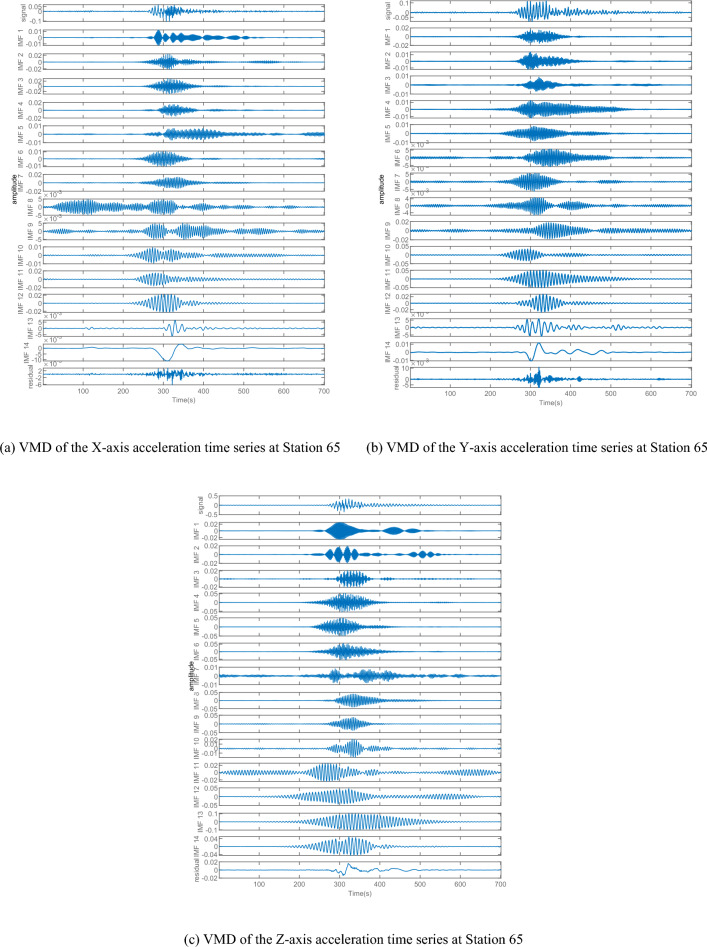
VMD of the acceleration time series at Station 65.

We employed the VMD method to eliminate noise from the accelerometer signal. The process involves decomposing the signal from high to low frequency via multiple layers and identifying noise mode components based on Flandrin denoise criterion. Following this, we added the remaining effective mode components to reconstruct the denoised signal successfully. Figure 16 illustrates the VMD decomposition signal of the Y-axis at station 65, where the decomposition level was set at 14 levels according to the spectrum's complexity and empirical values.

**Figure 17:**
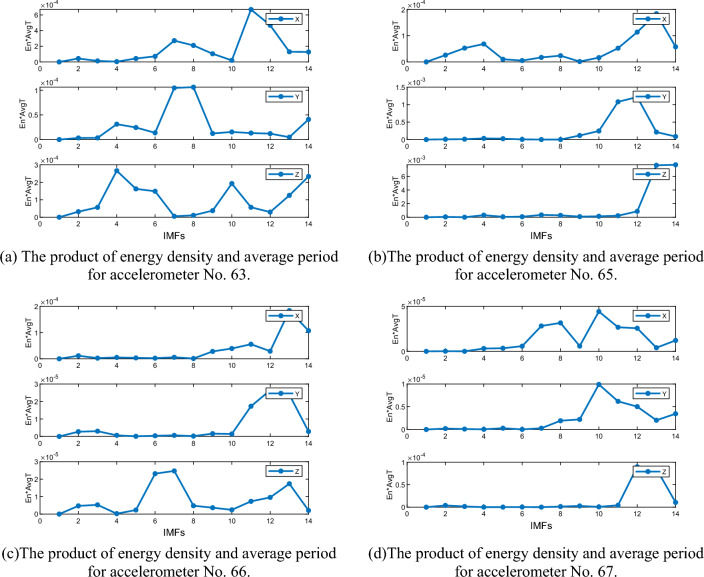
The product of energy density and average period for accelerometer.

The energy density and average period of numerical mutation points can serve as a standard for assessing noise components. Specifically, when ${ET}_{n}$ mutates, the preceding n-1 components $\left\{{\mu }_{1},{\mu }_{2}, {\mu }_{3}\dots {\mu }_{n-1}\right\}$ are considered noise components, while the following components $\left\{{\mu }_{n},{\mu }_{n+1}\dots {\mu }_{K}\right\}$ contain effective mode components with useful signals. Using these criteria and the product curve of energy density and average period for the three axes of the four monitoring stations (see in Fig. 17), we computed the effective mode components for each monitoring station and summarized them in Table 1.

**Figure 18:**
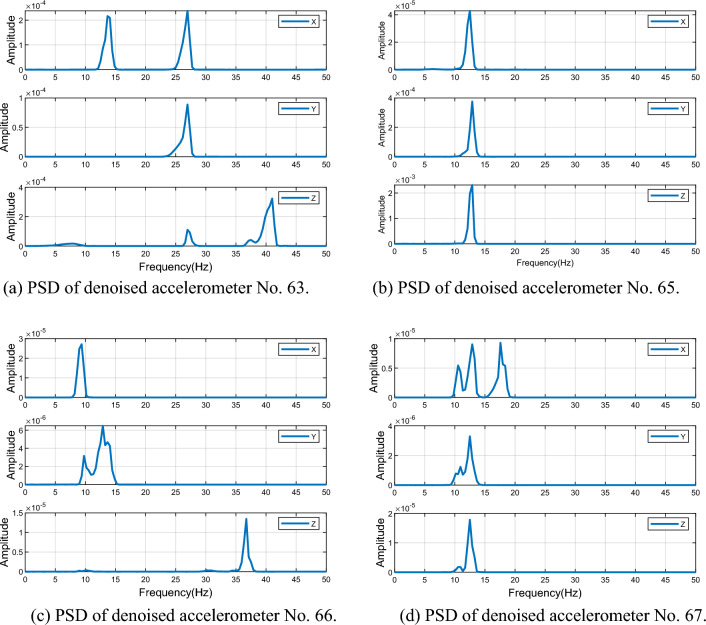
PSD of denoised accelerometer.

To reconstruct the acceleration signal, the effective mode components were summed up in Table 3 and the power spectral density curve was analyzed. The power spectral density curves of the four monitoring stations after denoising are presented in Fig. 18. It is evident that the power spectral density curve after denoising is smoother and the frequency is more identifiable than before denoising. For instance, station 65 exhibits only one frequency peak after denoising, with the extracted frequencies from the X, Y, and Z axes being 12.89 Hz, which can be attributed to earthquake excitation-induced vibration frequency. However, multiple frequency peaks still exist for stations 63, 66, and 67, and there are differences in the frequencies extracted from the X, Y, and Z axes. Therefore, it is not possible to accurately identify the vibration frequencies of stations 63, 66, and 67.

**Table 3:** Valid IMFs of acceleration time series of four monitoring stations.

Axis	No. 63	No. 65	No. 66	No. 67
X	7. 8. 11. 12	12. 13	13	7. 8. 10. 11
Y	7. 8	11. 12	11. 12. 13	10. 11. 12
Z	4. 5. 6. 10. 14	13. 14	6. 7. 13	12. 13

**Figure 19:**
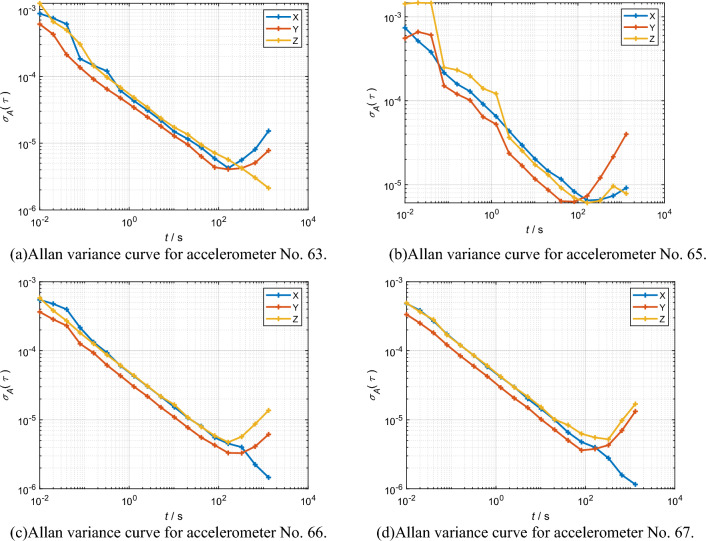
Allan variance curve for accelerometer.

To determine the reliability of vibration frequency data collected from stations 63, 66, and 67, the Allan variance method was employed to analyze the quality of data across all three axes. The performance of accelerometers is proportional to the proximity of their respective Allan variance curve to the lower left corner of an image and the tendency of their shape towards a V-shape or fish-hook shape. A comparison of Allan variance curves for different axes across four monitoring stations is presented in Fig. 19. It is observed that the Y-axis across all four stations has the highest reliability, as indicated by its proximity to the lower left corner and fish-hook shape. Conversely, the Z-axis of station 63, the X-axis of station 66, and the X-axis of station 67 have linear Allan variance curves, indicating unusable data. Therefore, the vibration frequencies extracted from the Y-axis of each monitoring station are deemed the most reliable and can be considered as the vibration frequency caused by earthquake excitation. The vibration frequencies at station 63, 65, 66, and 67 were measured to be 26.95 Hz, 12.89 Hz, 12.89 Hz, and 12.5 Hz, respectively (see in Table 4).

**Table 4:** Main frequencies of four monitoring stations.

Axis	No. 63 (Hz)	No. 65 (Hz)	No. 66 (Hz)	No. 67 (Hz)
X	13.67	12.5	9.38	12.89
				17.57
	26.95			
Y	26.95	12.89	12.89	12.5
Z	26.95	12.89	36.72	12.5
	41.02			

### Discussion

From the experimental results in Section “Acceleration time series analysis”, it can be seen that the accelerometers of all four monitoring stations were able to identify seismic events, indicating that the accelerometers used in this study have the ability to detect earthquakes. Of note, the detection times for earthquakes 66/67 were delayed by 0.5 s compared to 63/65, suggesting that this may be due to the greater distance between 66/67 and the epicenter compared to 63/65.

In Section “Spectrum analysis”, the spectral analysis of the raw accelerometer data revealed multiple peaks. After applying the VMD denoising method, the number of peaks decreased, but multiple peaks still remained. This is because the principle of VMD is to separate the signal into layers from high to low frequencies. When the frequency of the noise is close to the frequency of the seismic excitation signal, the VMD method still struggles to separate the noise. Furthermore, the frequency peaks of the three-axis accelerometers at each monitoring station were inconsistent, making it difficult to extract earthquake frequencies accurately.

To further improve the accuracy of earthquake frequency extraction, we introduced the Allan variance method. The Allan variance can detect the reliability and quality of accelerometer data and identify the most reliable set of acceleration data by analyzing the shape of the Allan variance curve. The spectral analysis results of this set of acceleration data are then used as the final earthquake frequency. However, the Allan variance method can only be used to determine which set of acceleration data among the three-axis accelerometers is the most reliable, narrowing down the range of earthquake frequency determination. It still cannot solve the problem of incomplete VMD effective mode component identification criteria leading to incomplete noise removal at the source.

## Conclusion

The accelerometer is a widely used seismic detection instrument. The observations from the accelerometer not only detect seismic events, but the spectral analysis results of the observations can also be used to extract earthquake frequencies. However, the original observations from accelerometer always contain noise components, resulting in PSD analysis results that include multiple peaks and making it difficult to determine the earthquake frequency. By combining VMD with noise component identification criteria, most of the noise can be removed, but residual noise still remains.

This research proposes an innovative method for extracting seismic excitation structural vibration frequencies by incorporating Allan variance, VMD, and PSD. This method introduces the Allan variance into vibration frequency extraction for the first time. By analyzing the Allan variance of the observations from the three-axis accelerometer, the unavailable observation can be eliminated while determining the most reliable set of observation. The peak frequency of the denoised spectral analysis of this set of observation is then considered as the final structural natural frequency.

The reliability of this method is demonstrated through analysis of vibration data collected by a three-axis accelerometer during the Mw2.6 Beijing Huairou microseism on August 2, 2022. Our experimental results indicate that the proposed approach can effectively detect microseisms of magnitude 2.6 and extract seismic excitation structural vibration frequency information. Specifically, we find that combining the Allan variance and VMD methods allows us to accurately extract seismic excitation vibration frequencies, which we report as 26.95 Hz, 12.89 Hz, 12.89 Hz, and 12.5 Hz for the four monitoring stations studied.

At present, the monitoring station provides only accelerometer data. To achieve more dependable spectral analysis outcomes, we intend to incorporate extra sensors, like gravimeters and GNSS, for comprehensive data fusion analysis. Furthermore, our future strategy includes collecting extensive monitoring data during seismic events to ascertain the reliability of the methodology proposed in this paper.

## Data Availability

The data that support the findings of this study are available from the Science and Technology Development Institute, Beijing University of Civil Engineering and Architecture. Restrictions apply to the availability of these data, which were used under license for the current study and so are not publicly available. However, data are available from the corresponding author, Xu Liu, should be contacted at liuxucrystal@163.com, upon reasonable request and with permission from the Science and Technology Development Institute.
